# How do *BRAF*^V600E^ and *TERT* promoter mutations interact with the ATA and TNM staging systems in thyroid cancer?

**DOI:** 10.3389/fendo.2023.1270796

**Published:** 2023-10-04

**Authors:** Noha Mukhtar, Kheloud Alhamoudi, Meshael Alswailem, Hindi Alhindi, Avaniyapuram Kannan Murugan, Balgees Alghamdi, Ali S. Alzahrani

**Affiliations:** ^1^Department of Medicine, King Faisal Specialist Hospital & Research Centre, Riyadh, Saudi Arabia; ^2^Department of Molecular Oncology, King Faisal Specialist Hospital & Research Centre, Riyadh, Saudi Arabia; ^3^Department of Pathology and Laboratory Medicine, King Faisal Specialist Hospital & Research Centre, Riyadh, Saudi Arabia

**Keywords:** thyroid cancer, differentiated thyroid cancer, *BRAF*^V600E^, *TERT* promoter mutations, risk stratifications

## Abstract

**Context:**

The American Thyroid Association risk stratification (ATA) and the American Joint Committee on Cancer Tumor Node Metastases (TNM) predict recurrence and mortality of differentiated thyroid cancer (DTC). *BRAF*^V600E^ and *TERT* promoter mutations have been shown to correlate with the histopathological features and outcome of DTC. Our objectives were to study the correlation of these molecular markers with these clinicopathological-staging systems.

**Patients and methods:**

We studied 296 unselected patients, 214 females and 82 males with a median age of 36 years (IQR 23.3-49.0). *BRAF*^V600E^ and *TERT* promoter mutations were tested by PCR-based Sanger sequencing. Data were extracted from medical records and analysed using Chi-Square and Fisher Exact tests and Kaplan Meier analysis.

**Results:**

Of 296 patients tested, 137 (46.3%) had *BRAF*^V600E^-positive tumors and 72 (24.3%) were positive for *TERT* promoter mutations. The *BRAF*^V600E^ mutation did not correlate with the ATA and TNM staging, being non-significantly different in various stages of these systems and did not predict the development of persistent disease (PD) (*P* 0.12). Unlike *BRAF*^V600E^, *TERT* promoter mutations were more frequent in the ATA high-risk than in intermediate- or low-risk tumors (P 0.006) and in TNM stages III and IV than lower stages (*P <*0.0001). *TERT* promoter mutations also predicted the outcome, being present in 37.2% of patients with PD compared to only 15.4% in those without evidence of disease (*P <*0.0001). The same pattern was also seen when *BRAF*^V600E^ and *TERT* promoter mutations were combined.

**Conclusion:**

*TERT* promoter mutations alone or in combination with *BRAF*^V600E^ mutation, but not *BRAF*^V600E^ mutation alone, correlated well with the ATA and TNM staging and predicted development of PD, especially in higher stages of these systems.

## 1 Introduction

Differentiated thyroid cancer (DTC) has been increasingly diagnosed over the past four decades (1). This increase in incidence is largely attributable to improved diagnostic tools, particularly the widespread use of neck ultrasonography (1, 2). As a result, many small tumors with low risk of metastasis, recurrence, and mortality have been detected (3). This fact led to a more conservative approach to the management of DTC based on risk stratification (4, 5). Currently, several risk stratification systems are available for risk-based management planning (6–9). Two of the most widely used systems are the American Thyroid Association (ATA) and the American Joint Committee on Cancer Tumor Node and Metastasis (AJCC TNM) risk stratification systems (7, 8). The ATA system predicts risk of DTC recurrence, while the TNM system predicts risk of DTC-related mortality (6, 7, 9). Both systems have been well validated and are routinely used in clinical practice and research communications (9–11).

Advances in understanding the molecular pathogenesis of DTC have paralleled better understanding of its clinical behavior and outcome and the development of risk stratification systems (12–17). These advances in molecular genetics led to development of diagnostic tests and therapeutic agents for patients with thyroid nodules and advanced thyroid cancer, respectively (14, 18–20). The use of molecular markers for prognostication of DTC has also been studied but remains controversial and less mature than diagnostic and therapeutic advances (7, 21, 22). One of the earliest discoveries is the *BRAF*^V600E^ mutation as a major oncogenic driver in papillary thyroid cancer (PTC) and to a lesser extent in poorly differentiated (PDTC) and anaplastic thyroid cancer (ATC) (15, 23, 24). Several studies have shown a strong association between *BRAF*^V600E^ mutation and aggressive histopathological features of DTC (25–28). However, others questioned its prognostic value (29, 30). More recently, *TERT* promoter mutations (C250T and C228T) were discovered as strong oncogenic drivers in many types of thyroid cancer (31–33). They occur in approximately 10% of well-differentiated PTC but are increasingly commoner in the more aggressive types such as PDTC and ATC (13). Although these mutations are associated with aggressive histopathological features and worse outcome of DTC, especially when they co-occur with *BRAF*^V600E^ or *RAS* mutations (34, 35), their use as prognostic markers is not yet widely accepted (7). The 2015 ATA guidelines acknowledge the potential prognostic value of *BRAF*^V600E^ and *TERT* promoter mutations but do not fully endorse it or routinely recommend it (7). To further study the potential relationship between the clinicopathological staging systems and the driver mutations of DTC, we hypothesized that these mutations are more prevalent in higher ATA and TNM stages than the low-risk stages and that they may contribute further to risk stratification in different stages of these systems. For these reasons, we studied a cohort of patients with DTC in whom *BRAF*^V600E^ and *TERT* promoter mutations have been tested and assessed their relationships with the ATA and TNM risk stratification systems. Specifically, we assessed the prevalence of *BRAF*^V600E^ and *TERT* promoter mutations in different ATA and TNM stages and analysed their potential incremental prognostic value over these risk stratification systems.

## 2 Patients and methods

An Institutional Review Board (IRB) approval was obtained from the Office of Research Affairs, King Faisal Specialist Hospital and Research Centre, Riyadh Saudi Arabia (ORA # 2020-1514) with a waiver of consent to use archived Formalin Fixed Paraffin Embedded (FFPE) samples for mutation testing. We isolated tumor DNA, performed PCR and directly sequenced exon 15 of *BRAF* gene and the *TERT* promoter using the Dideoxy Chain Termination method. The DNA isolation, PCR primers and conditions, and the Sanger sequencing methods for *BRAF*^V600E^ and *TERT* promoter mutations have been previously described (36–38). A total of 296 unselected DTC patients in whom *BRAF*^V600E^ and *TERT* promoter mutations were available have been included in this study. Data on their demographics, histopathological data, ATA and TNM staging, management and outcome were obtained from their medical records. The outcome was assessed based on definitions included in the 2015 ATA guidelines (7). An excellent response (175 patients) was defined as absence of any evidence of disease with suppressed serum thyroglobulin (Tg) < 0.2 ng/dl and/or stimulated Tg < 1 ng/dl in the absence of Tg antibodies and negative imaging studies. Persistent disease included patients with structurally incomplete, biochemically incomplete and indeterminate response to therapy statuses as defined in the ATA guidelines for DTC (7).

### 2.1 Statistical methods

We expressed continuous variables as medians and interquartile ranges and categorical variables as rates, proportions and percentages. Fisher Exact and X^2^- tests were used for analysis of categorical variables and T test for continuous variables. Kaplan Meier survival analysis was used to analyse outcome over time stratified by ATA or TNM stages or presence or absence of *BRAF*^V600E^ mutation and/or *TERT* promoter mutations. Disease-free survival is the time between the initial thyroid surgery and diagnosis of indeterminate, biochemically or structurally incomplete response (evidence of disease). A *P* value < 0.05 was considered significant.

## 3 Results

### 3.1 Clinicopathological characteristics

We studied 296 patients, 214 (73.3%) females, 82 (27.7%) males (F:M ratio 2.6:1) with a median age of 36 years (IQR 23.25-49 years). *BRAF*^V600E^ mutation was significantly more prevalent in patients ≥ 55 years (29/45, 62%) than in those less than 55 years of age (109/251, 43.3%), *P* 0.03. Similarly, *TERT* promoter mutations were more prevalent in patients ≥ 55 years (28/45, 64.4%) than in those less than 55 years of age (43/251, 17%), *P <*0.0001. The histopathological characteristics, management and outcome are summarized in **Table 1**. The median follow up was 7.6 years (Interquartile range 5.25-10.1)

**Table 1:** The clinical and pathological features, staging and outcome of 296 DTC patients.

Characteristic	Median and IQR or No. (%)
Median age (IQR), years	36 (23.25-49)
Sex F:M	214:82
**Tumor type** Classic papillary thyroid cancer (PTC) Follicular variant PTC Tall cell variant PTC Diffuse sclerosing PTC Follicular thyroid cancer Oncocytic (Hurthle) cell cancer	181 (61) 62 (21) 34 (11.5) 8 (2.7) 7 (2.4) 4 (1.4)
Median Tumor size (range) cm	2.5 (1.65-4.5)
Tumor multifocality	188 (63.5)
Extra-thyroidal extension	152 (51.4)
Lymphovascular invasion	97 (32.8)
Lymph node metastases	192 (64.9)
Distant metastases	47 (15.9)
**ATA risk stratification** Low risk Intermediate Risk High risk	96 (32.4) 125 (42.2) 75 (25.3
**TNM stages** I II III IV	99 (33.4) 150 (50.7) 27 (9.1) 20 (6.8)
Received radioactive iodine-131	259 (87.5%)
Median administered activity	123 mCi (100-150)
Received additional therapies	95 (32%)
**Outcome** No evidence of disease (Excellent response) Persistent disease (Indeterminate response, biochemically and structurally incomplete)	175 (59) 121 (41)

### 3.2 ATA and TNM risk stratification systems predict outcome of DTC

**Figure 1:**
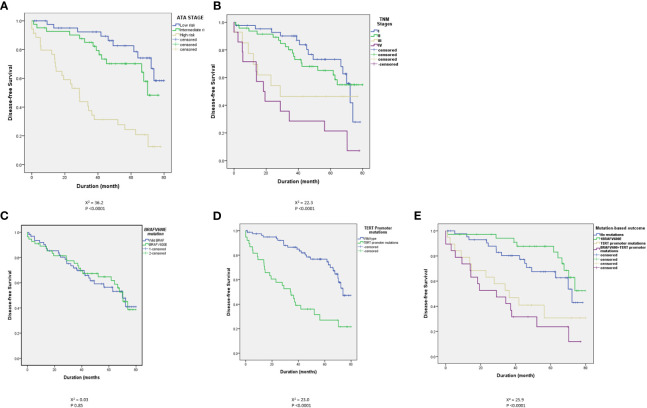
Kaplan Meier curves showing a clear and significant separation of disease-free survival (DFS) between different ATA stages **(A)** and different TNM stages **(B)**. There was no statistically significant difference between *BRAF*^V600E^-positive and wild *BRAF* tumors **(C)** but there are statistically significant differences between *TERT* promoter mutation-positive tumors and tumors with wild type *TERT* promoter in the absence **(D)** or presence **(E)** of concomitant *BRAF*^V600E^ mutation.

As demonstrated in many previous studies, in this study, the ATA and TNM risk stratification systems predict the outcome (**Table 2** and **Figure 1**). Persistent disease increases from 19.8% in ATA low-risk to 72% in the high-risk classes (P<0.0001). Similarly, persistent disease increases from 31.3% in TNM stage I to 95% in stage IV (p <0.0001), (**Table 2**). Kaplan Meier analysis shows significant differences in the disease-free survival between different ATA and TNM stages (**Figure 1**). Of six patients who died due to DTC, five were ATA high grade and one ATA intermediate grade. Two were in TNM stage 2, two in stage 3 and two in stage 4.

**Table 2:** Outcome of DTC in different ATA and TNM stages showing more persistent thyroid cancer in higher stages.

ATA risk group	NED No. (%)	PD No. (%)	*P* value
Low risk	77 (80.2)	19 (19.8)	<0.0001
Intermediate risk	77 (64)	48 (38.4)	
High risk	21 (28.0)	54 (72.0)	
TNM stages			
I	68 (68.7)	31 (31.3)	<0.001
II	97 (64.7)	53 (35.3)	
III	9 (33.3)	18 (66.7)	
IV	1 (5)	19 (95)	
**Total**	**175 (59)**	**121 (41)**	

NED, no evidence of disease; PD, persistent disease.

### 3.3 *BRAF*^V600E^ mutation does not correlate with ATA and TNM staging

Of 296 patients tested, 137 (46.3%) had *BRAF*^V600E^-positive tumors while 159 patients (53.7%) had wild-*BRAF* tumors. *BRAF*^V600E^ was positive in 92 of 181 (50.8%) conventional PTC, 18 of 62 (29%) follicular variant PTC (FVPTC), 25 of 34 (73.5%) tall cell varia PTC (TCPTC) and 2 of 8 (25%) diffuse sclerosing PTC (DSPTC). *BRAF*^V600E^ mutation did not correlate with the ATA and TNM staging. As seen in **Table 3**, the rates of the mutation were not significantly different between low and higher stages. *BRAF*^V600E^ also did not predict the outcome (**Table 4** and **Figure 1**) with no difference in the rates of *BRAF*^V600E^ mutation between those who achieved an excellent response (42.3%) and those who had a persistent disease (52.1%) (*P* 0.12), (**Table 4**). Six patients died of DTC in this cohort, four of them were *BRAF*^V600E^ mutation-positive.

**Table 3:** The prevalence of *BRAF*^V600E^ and *TERT* promoter mutations in different ATA and TNM stages.

	Wild *BRAF* No. (%)	*BRAF*^V600E^ No. (%)	P value	Wild *TERT* No. (%)	*TERT* promoter No. (%)	P value
ATA staging						
Low risk	54 (56.2)	42 (43.8)	0.33	74 (77.1)	22 (22.9)	0.007
Intermediate risk	61 (48.8)	64 (51.2)		103 (82.4)	22 (17.6)	
High risk	44 (58.7)	31 (41.3)		47 (62.7)	28 (37.3)	
TNM staging						
I	55 (55.6)	44 (44.4)	0.41	96 (97.0)	3 (3.0)	<0.001
II	77 (51.3)	73 (48.7)		104 (69.3)	46 (30.7)	
III	18 (66.7)	9 (33.3)		19 (70.4)	8 (29.6)	
IV	9 (45)	11 (55)		5 (25)	15 (75)	

**Table 4:** Outcome of DTC with respect to *BRAF*^V600E^, *TERT* promoter mutations or both.

	Total No. (%)	NED No. (%)	PD No. (%)	P value
*BRAF*^V600E^ mutation	137 (46.3)	74/175 (42.3)	63/121 (52.1)	0.12
*TERT* promoter mutations	72 (24.3)	27/175 (15.4)	45/121 (37.2)	<0.0001
*BRAF*^V600E^+*TERT* promoter mutations	34 (11.5)	8/175 (4.9)	26/121 (21.5)	<0.0001

NED, no evidence of disease; PD, persistent disease.

### 3.4 *TERT* promoter mutations correlate with the ATA and TNM staging and predict outcome

*TERT* promoter mutations, C250T (9 tumors) and C228T (63 tumors) were found in tumors of 72 patients (24.3%). *TERT* promoter mutations were positive in 40/181 (22.1%) conventional PTC, 17/62 (27.4%) FVPTC, 9/34 (26.5%) TCPTC, 2/8 (25%) DSPTC, 3/7 (43%) follicular thyroid cancer (FTC) and ¼ (25%) Oncocytic thyroid cancer. Unlike *BRAF*^V600E^ mutations, *TERT* promoter mutations were more frequent in the ATA high-risk (37.3%) than in intermediate- (17.6%) or low-risk tumors (22.9%) (*P* 0.007) (**Table 3**). More clearly is the higher prevalence of *TERT* promoter mutations in TNM stage IV (75%) than lower stages (**Table 3**). *TERT* promoter mutations also predicted the outcome, being present in 37.2% of patients with persistent disease compared to only 15.4% in those without evidence of disease (*P <*0.0001) (**Table 4** and **Figure 1**). *TERT* promoter mutations were significantly more frequent in patients with structurally incomplete disease than other response to therapy status groups, being positive in only 19/175 (10.9%) in excellent response, 3/54 (5.6%) of indeterminate response, 3/14 (21.4%) of biochemically incomplete and 13/53 (24.5%) of structurally incomplete response (P <0.0001) (**Table 5**). Of six patients who died due to DTC, five (83%) were positive for *TERT* promoter mutations.

**Table 5:** The outcome of 296 patients with DTC and its relationship to *BRAF*^V600^ and *TERT* promoter mutations.

	Excellent response No. (%)	Indeterminate response No. (%)	Biochemically incomplete No. (%)	Structurally incomplete No. (%)	P value
**BRAF Wild type**	101 (57.7)	23 (42.6)	5 (35.7)	30 (56.6)	0.12
***BRAF*^V600E^**	74 (42.3)	31 (57.4)	9 (64.3)	23 (43.4)	
***TERT* wild type**	148 (84.6)	46 (85.2)	5 (35.7)	25 (47.2)	<0.0001
***TERT* promoter mutated**	27 (15.4)	8 (14.8)	9 (64.3)	28 (52.8)	
**Total**	**175**	**54**	**14**	**53**	**296**

In a multivariate logistic regression model that included *BRAF*^V600E^ mutation, *TERT* promoter mutations, age at diagnosis, tumor size, ATA stage and TNM stage, *TERT* promoter mutations remain a significant predictor of persistent disease (*P* 0.01, odds ratio 2.7, 95% CI 1.2-5.9).

### 3.5 Combination of *BRAF*^V600E^ and TERT promoter mutations correlate with the ATA and TNM staging and predict outcome of DTC

The combination of *BRAF*^V600E^ mutation and a *TERT* promoter mutation occurred in 34 cases (11.5%) and correlated well with high-risk ATA and higher TNM stages (**Table 6**). This combination occurred in 21.3% in high-risk ATA class compared to 8.8% and 7.3% in intermediate and low-risk stages, respectively (*P* 0.006, **Table 6**). It also occurred in 45% of TNM stage IV compared to 7.4%, 14.7% and 1% in stages III, II, and I, respectively (*P* < 0.0001) (**Table 6**). Of 34 cases that had this combination of *BRAF*^V600E^/mutated *TERT*, 26 (76.5%) continued to have persistent disease compared to only 8 (23.5%) in excellent response (P <0.0001) (**Table 4**). The percentages of patients with persistent disease increased progressively from 12.8% in patients with ATA low-risk and no mutations to 100% in patients with ATA high-risk with positive *BRAF*^V600E^/*TERT* promoter mutations (**Table 7**). Similarly, persistent disease increased progressively from 26.4% in stage I without *BRAF*^V600E^ or *TERT* promoter mutations to 100% in stage IV disease with positive *BRAF*^V600E^/*TERT* promoter mutations (**Table 7**). In fact, all patients (100%) with *BRAF*^V600E^ and *TERT* promoter mutation combination who are in ATA high-risk or TNM stage IV groups continued to have persistent disease (**Table 7**). The combination of *TERT* promoter/*BRAF*^V600E^ mutations were significantly more frequent in patients with biochemically and structurally incomplete disease than other response to therapy status groups being positive in only 8/175 (4.6%) in excellent response, 5/54 (9.3%) of indeterminate response, 6/14 (42.9%) of biochemically incomplete and 15/53 (28.3%) of structurally incomplete response (P <0.0001).

**Table 6:** Rates of different combinations of *BRAF*^V600E^ and *TERT* promoter mutations in different ATA and TNM stages.

Stage (Number of patients)	Wild *BRAF*/ wild *TERT* No. (%)	*BRAF*^V600E^/ wild *TERT* No. (%)	Wild *BRAF*/ Mutated *TERT* No. (%)	*BRAF*^V600E^/ mutated *TERT* No. (%)	P value
ATA staging					
Low-risk (96)	39 (40.6)	35 (36.5)	15 (15.6)	7 (7.3)	0.006
Intermediate risk (125)	50 (40.0)	53 (42.4)	11 (8.8)	11 (8.8)	
High-risk (75)	32 (42.7)	15 (20.0)	12 (16)	16 (21.3)	
TNM staging					
I (99)	53 (53.5)	43 (43.4)	2 (2.0)	1 (1.0)	<0.0001
II (150)	53 (35.3)	51 (34.0)	24 (16.0)	22 (14.7)	
III (27)	12 (44.4)	7 (25.9)	6 (22.2)	2 (7.4)	
IV (20)	3 (15.0)	2 (10.0)	6 (30)	9 (45.0)	

**Table 7:** Number of cases with persistent disease/total number (%) in each ATA and TNM stage categorized by the presence and type of mutation.

Stage	No mutations No. (%)	*BRAF only* No. (%)	*TERT only* No. (%)	*BRAF/TERT* No. (%)	Total
ATA stages					P <0.0001
Low	5/39 (12.8)	9/35 (25.7)	3/15 (20)	2/7 (28.6)	19/96 (19.8)
Intermediate	16/50 (32.0)	20/53 (37.7)	4/11 (36.4)	8/11 (72.7)	48/125 (38.4)
High	18/32 (56.2)	8/15 (53.3)	12/12 (100)	16/16 (100)	54/75 (72.0)
TNM stage					P <0.0001
I	14/39 (26.4)	16/43 (37.0)	1/2 (50.0)	0/1 (0)	31/99 (31.3)
II	15/53 (28.5)	16/35 (31.4)	7/24 (29.2)	15/22 (68.5)	53/150 (35.3)
III	7/12 (58.3)	4/7 (57.1)	5/6 (83.3)	2/2 (100)	18/27 (66.7)
IV	3/3 (100)	1/2 (50.0)	6/6 (100)	9/9 (100)	19/20 (95)

## 4 Discussion

The ATA and TNM staging systems predict risk of recurrence and mortality, respectively (7–9). *BRAF*^V600E^ and *TERT* promoter mutations have also been shown to predict risk of recurrence and mortality (26, 33, 39, 40). However, the relationship between these histopathological systems and molecular markers is not clear. In this study, we tried to analyse this potential relationship. Our findings confirm the previously shown high accuracy of the ATA and TNM staging systems in predicting the outcome (persistent disease) (7, 9) and lack of an association between *BRAF*^V600E^ and these staging systems and the DTC outcome. However, the main finding of this study is the strong association between *TERT* promoter mutations and the ATA and TNM staging systems, and the high prognostic value of these mutations in isolation or in combination with *BRAF*^V600E^ mutation in predicting the outcome of DTC, especially in the high ATA and TNM stages. The occurrence of these mutations in ATA high-risk or TNM stage IV tumors was associated with 100% chance of persistent disease. This suggests that *TERT* promoter mutations± *BRAF*^V600E^ mutation identify a subgroup of patients in the high-risk ATA or TNM who have an extremely high risk of persistent disease. This group may need more proactive management and follow up approaches.

The association between *TERT* promoter mutations and aggressive histopathological features and outcome of DTC has been reported in several studies from different parts of the World. Similar to our study, a meta-analysis that included 11 studies and 3911 patients showed a graded risk of DTC based on the presence or absence of *TERT* promoter mutations and *BRAF*^V600E^ mutation with the highest risk in DTC harboring both types of mutations followed by DTC with *TERT* mutation alone, *BRAF*^V600E^ mutation and no mutation (41). In a more recent meta-analysis that included 51 studies with 11,382 patients from different populations, *TERT* promoter mutations were found in 10.9% of DTC in general and in 10.6% of PTC and 15.1% of FTC. In PTC, *TERT* promoter mutations were significantly associated with sex, age, tumor size, vascular invasion, extrathyroidal extension, lymph node and distant metastases, persistence/recurrence, and disease-specific mortality. Similarly, in FTC, *TERT* promoter mutations were significantly associated with age, distant metastases, advanced TNM stage, persistence/recurrence, and disease-specific mortality (42).

In another recent meta-analysis that looked at risk factors for development of radioiodine refractory thyroid cancer (RAIR), Luo Y. et al. included 13 studies with 1431 patients, of whom 603 were patients with RAIR. *TERT* and *BRAF*^V600E^ mutations, extrathyroidal extensions and high-grade histopathological thyroid cancer subtypes were associated with increased risk of development of RAIR (43).

Over the past 3 decades, several staging systems have been proposed and validated (6, 8, 9). Although most of the old systems were designed to predict mortality of DTC, mortality is very low in DTC (2). On the other hand, persistent/recurrent DTC is common occurring in approximately 20-30% of patients (7, 44). Currently, the ATA risk stratification system, which encompasses several histopathological tumor features, is the most widely used system for predicting recurrence of DTC (7). It considers risk of DTC recurrence as a continuum but also classifies DTC into low-, intermediate- and high risk for recurrence (7, 45). Several studies have shown the robustness of this system for predicting recurrence and it is currently the most widely used system in clinical practice and research communication (10, 11, 46, 47). The AJCC TNM system is one of the mortality-predicting staging systems and is based on age and several histopathological features including tumor size, extrathyroidal invasion, lymph node and distant metastasis (8, 48). It has also been shown to be highly reliable in predicting cancer-specific mortality (8, 48).

The significant progress that took place in the field of molecular genetics of DTC was also translated in clinical practice to diagnostic tests for indeterminate thyroid nodules and therapeutic agents for progressive radioactive iodine refractory thyroid cancer (49–51). Due to conflicting studies and variable behavior of DTC carrying *BRAF*^V600E^ or *TERT* promoter mutations, the use of these genetic markers in predicting the course and outcome of DTC remain controversial (7, 22, 52). In fact, the 2015 ATA thyroid cancer guidelines acknowledged the potential roles of these genetic markers for prognostication but did not fully endorse them as a basis for intensity of the management and follow up of patients with DTC (7).

Since the ATA and TNM staging systems are clinicopathological systems for predicting the outcome and *BRAF*^V600E^ and *TERT* promoter mutations are potential predictors of outcome, we undertook this study to assess any potential relationship between these clinicopathological and molecular predictors of prognosis. Specifically, we aimed to study whether *BRAF*^V600E^ and/or *TERT* promoter mutations may add incremental prognostic value to the ATA and TNM staging systems. Our results suggest that *BRAF*^V600E^ does not correlate with the ATA or TNM staging systems and does not predict the outcome alone. However, *TERT* promoter mutations alone or in combination with *BRAF*^V600E^ mutation have significant correlation with the ATA and TNM risk stratification systems and are predictive of disease-free survival and persistent/recurrent disease, especially in high-stage DTC. In patients with ATA high-risk group and TNM stage IV, the presence of *TERT* promoter alone or in combination with *BRAF*^V600E^ predicts a very high probability of persistent disease. These results are in agreement with several studies that have shown a strong impact of *TERT* promoter mutations on the DTC behavior and outcome, especially when they co-occur with *BRAF*^V600E^ mutation (33–36). However, our study also joins several previous studies that casted doubts on the prognostic role of *BRAF*^V600E^ mutation alone (21, 29, 30, 39, 53). While there is no doubt about the strong oncogenic role of *BRAF*^V600E^, its final impact on the DTC behavior is probably influenced by other histopathological features and the stage of the disease (39, 53). The strong synergistic effect of *TERT* promoter mutations on tumors that also harbor *BRAF*^V600E^ mutation is clear (34, 35) and it is possible that old studies that showed a strong prognostic impact of *BRAF*^V600E^ mutation were enriched by then the unknown *TERT* promoter mutations. In other words, it is possible that studies that showed a strong prognostic role of *BRAF*^V600E^ had high rates of *TERT* promoter mutations, which were not known to occur in DTC at the time of these old studies before 2013.

Our study has strengths and weaknesses. It included a good sample size from a single institution with uniform practice. However, the sample size is still relatively small for the study of an association. Reassuring in this study about the sample representation of DTC is the fact that the patients’ characteristics, the histopathological features, the rates of *BRAF*^V600E^ and *TERT* promoter mutations and the distribution of patients between different ATA and TNM risk classes are the usual spectrum of DTC seen in most centers. The clinic pathological features are similar to a previous descriptive study in which we characterized DTC in Saudi population (54). Notably, the median age in our population (36 years) is younger than the median age of the SEER data (51 years) and the rate of distant metastases is high. These are similar in this study to our previous publication (54) and a more comprehensive recent study that looked at thyroid cancer in Saudi Arabia over the last 30 years (55). The rate of *TERT* promoter mutations is also relatively higher in our study than The Cancer Genome Atlas (TCGA) database but this latter contained only well differentiated PTC and our study contained a significant number of patients with tall cell subtype of PTC and other DTC types accounting for the relatively high *TERT* promoter mutation.

In summary, we have shown that *BRAF*^V600E^ alone does not correlate with the widely used ATA and TNM staging systems while *TERT* promoter mutations alone or in combination with *BRAF*^V600E^ do correlate with these systems and predict DTC outcome. Their presence in higher stages of these risk stratification systems is associated with a very high risk of persistent disease and probably worse outcome. Further studies with larger sample size, preferably multi institutional, are needed to assess the incremental prognostic value of these molecular markers over the current ATA and TNM risk stratification systems.

## Data availability statement

The original contributions presented in the study are included in the article/supplementary material. Further inquiries can be directed to the corresponding author.

## Ethics statement

The studies involving humans were approved by Office of Research Affairs, King Faisal Specialist Hospital & Research Centre, Riyadh, Saudi Arabia. The studies were conducted in accordance with the local legislation and institutional requirements. The ethics committee/institutional review board waived the requirement of written informed consent for participation from the participants or the participants’ legal guardians/next of kin because we only used archived paraffin block without any direct contact with the patients and the data were kept anonymous.

## Author contributions

NM: Data curation, Writing – review & editing. KA: Data curation, Writing – review & editing. MA: Data curation, Methodology, Writing – review & editing. HA-H: Methodology, Writing – review & editing, Resources. AM: Data curation, Writing – review & editing. BA: Data curation, Methodology, Writing – review & editing. AA: Methodology, Conceptualization, Formal Analysis, Writing – original draft.
